# Modeling analysis and lightweight design for an axle hub considering stress and fatigue life

**DOI:** 10.1177/0036850420929930

**Published:** 2020-08-02

**Authors:** Cuixia Zhang, Quande Dong, Liangxi Zhang, Qiang Li, Jianqing Chen, Xuehong Shen

**Affiliations:** 1 School of Mechanical and Electronic Engineering, Suzhou University, Suzhou, China; 2 School of Information Engineering, Suzhou University, Suzhou, China; 3 QiuZhen School, Huzhou University, Huzhou, China; 4 Xiangyang Road and Bridge Construction Group Co., Ltd., Xiangyang, China

**Keywords:** Lightweight design, stress, fatigue life, ANSYS, energy conservation

## Abstract

The lightweight of axle hub has been recognized as a typical representative of reducing the quality of vehicles for energy saving and emission reduction. Therefore, a modeling analysis and lightweight design based on ANSYS for axle hub is proposed. Firstly, design dimensions and structural parameters of axle hub are modeled. The hub model is imported into workbench software to simulate the static analysis. The stress and deformation of the axle hub are obtained by mesh element division, boundary condition constraints and loading conditions, and the modal and fatigue strength of the hub are simulated to obtain the modal natural frequency and life data. Then, from the point of view of reducing the quality and safety of the hub, the structure of the hub is changed, the model is compared again, and the lightweight design scheme can be obtained. The results show that the weight of a hub decreases by 5.537% after lightening. This method can improve the structure, save materials, reduce production costs, and shorten the design cycle. This lightweight design method has important reference and practical significance for the lightweight design of axle wheels and similar products.

## Introduction

Since the 21st century, the global resource crisis has become a difficult problem for the sustainable development of manufacturing industry.^1,2^ Therefore, the lightweight design of mechanical products has become the focus of attention in the process of enterprise research and development.^3^ Lightweight design is considered to play an important role in improving equipment performance and reducing cost.^4^ The axle hub is a key component of transportation equipment. Therefore, its excellent lightweight has been recognized as a great significance to the safety, cost, quality, and energy saving. The lightweight design of axle hub not only considers the stiffness, stress, and safety factor, but also ensures the safety, reliability, and stability of vehicle in the course of running.^5^ Many experts and scholars have studied the lightweight design of parts and components. Topouris and Tirovic^6^ researched on lightweight designed a monobloc fingered hub to reduce disk mass, maintain rotor thermal capacity, and improve heat dissipation characteristics. Li et al.^7^ studied six-sigma robust design optimization to explore the lightweight design and crashworthiness of electric vehicles with uncertainty. Deng et al.^8^ researched on the life cycle optimization design of lightweight components of automobiles with flax fiber–reinforced polymer composite. Yin et al.^9^ proposed the lightweight design of a lightweight manipulator hybrid structure based on carbon fiber–reinforced plastics and aluminum alloy. Liang et al.^10^ studied the lightweight design of automotive front rails by nonlinear structural optimization method. Ma et al.^11^ aimed for devising a concise formulation for lightweight optimal design of bolted joint systems without gaskets. Zhang et al.^12^ researched on lightweight design of heavy vehicle transmission and action components. Croccolo et al.^13^ addressed the design and optimization of interference-fit and adhesively bonded joints in lightweight structures for shaft–hub.

These studies have greatly promoted the development of lightweight design of automotive parts. Based on the above studies,^14,15^ a modeling analysis and lightweight design based on ANSYS for axle hub is proposed considering stress and fatigue life. This article provides both theoretical and practical contributions. In theory, on the basis of its structural analysis, the modal, fatigue strength, and life of the hub are simulated by finite element method, and the lightweight design of the hub is verified. This method provides a useful reference for the lightweight design of automotive parts. In engineering practice, this method can clearly tell the engineering designers how to efficiently and quickly lighten the mechanical parts of automobile. It can improve the structure, save materials, reduce production costs, shorten the design cycle, and meet the demand of customers for manufacturing enterprises.

## Methods

### ANSYS model and static analysis

In this article, the three-dimensional model of the hub is built by Pro/E software, and then introduced to static structural module of Workbench.

**Figure 1.**
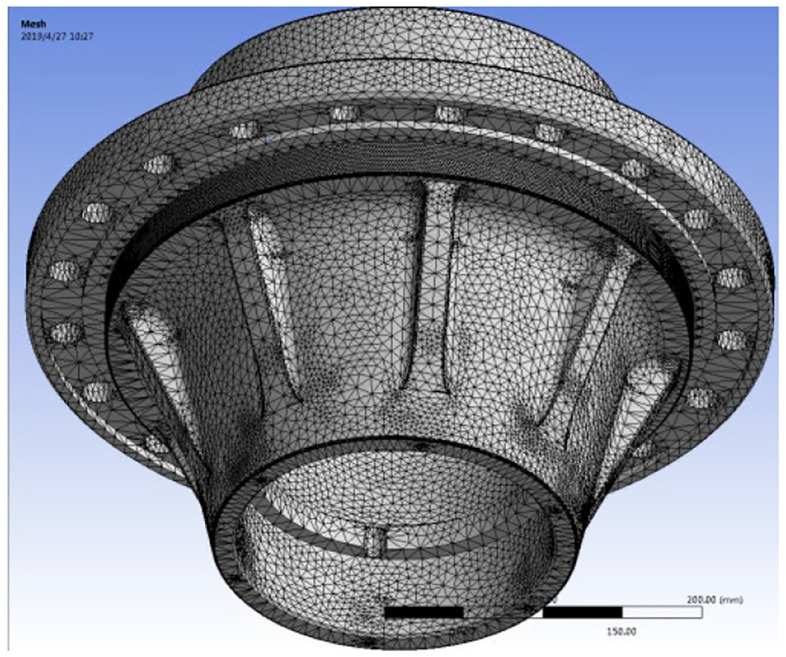
Mesh generation of hub model.

Because the hub is a complex multi-surface model, this analysis chooses three-dimensional solid tetrahedron and local refinement of the hub stress surface to divide the mesh (as shown in Figure 1). After dividing, the solid element is 311,023, and the number of mesh nodes is 523,341.

**Figure 2.**
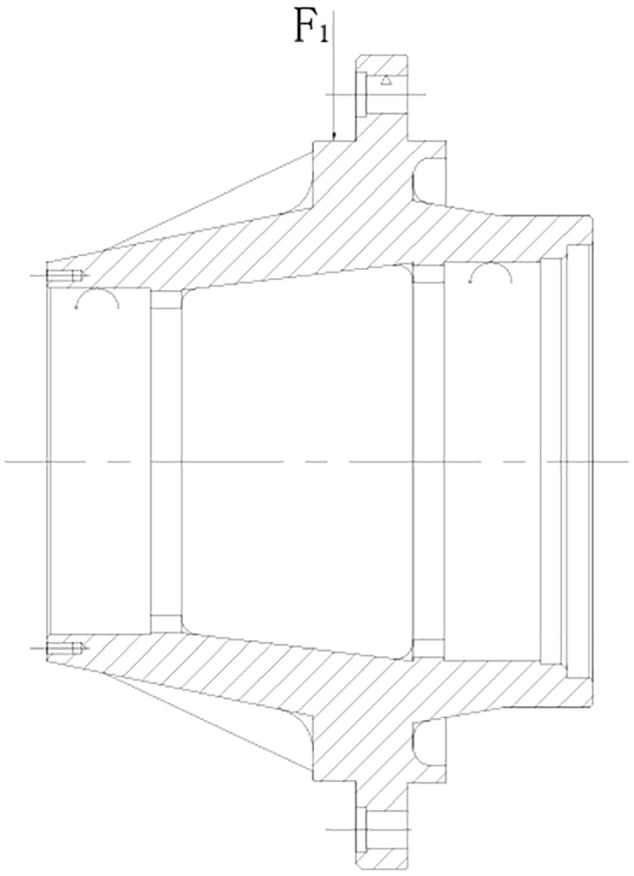
Schematic diagram of hub under load and constraints.

Wheel hub is connected with brake drum and hub bearing, which ensures that the wheel is securely fixed on the vehicle. The restrained movement is mainly restricted by the steel ring on the wheel tire. It imposes fixed constraints on the tire bolts on the flange of the hub, and cylindrical constraints on the two hub bearings on the inner side of the hub, thus restricting the movement of the hub in the tangential direction of the axle, but it can rotate. Because the hub structure is mainly affected by the restraint load imposed on the rabbet, the impact load $F_{1}$ acts on the rabbet as shown in Figure 2.

Impact load force $F_{1}$ of hub rabbet face

$$F_{1}=k\times G=1.2\times 3.0\times 10^{5}N=3.6\times 10^{5}N$$

where *K* is the impact load coefficient, *G* is the vehicle mass load.

Working stress^16^ of hub *σ*

$$\sigma =\frac{F_{1}}{S}=\frac{F_{1}}{\pi \mathrm{Dh}}=\frac{3.6\times 10^{5}N}{\pi \times 370\times 10^{-3}\times 25\times 10^{-3}}\mathrm{Pa}=12.388\;\mathrm{MPa}$$

where S is the force area of the hub.

Allowable stress^17^ of materials [σ]

$$\left[\sigma \right]=\frac{S_{\mathrm{Yt}}}{n_{s}}=\frac{3.6\times 10^{8}}{2.0}=180\;\mathrm{MPa}$$

where $n_{s}$ is the safety factor of material.

From the above calculation, we can see that

σ=12.388MPa≤[σ]=180MPa

Through the above strength check, it is known that the stress condition of the hub meets the safety requirements.

According to the boundary condition management of the hub, the equivalent force, displacement, and safety factor of the hub under load are simulated.

**Figure 3.**
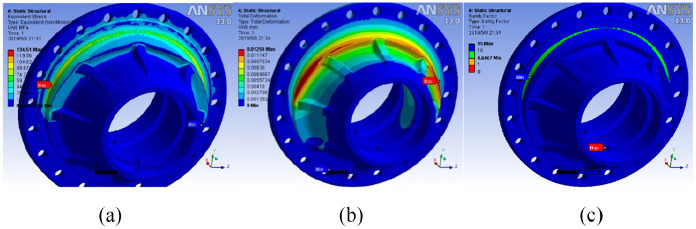
(a) Stress, (b) displacement, and (c) safety factor distribution of hub.

**Figure 4.**
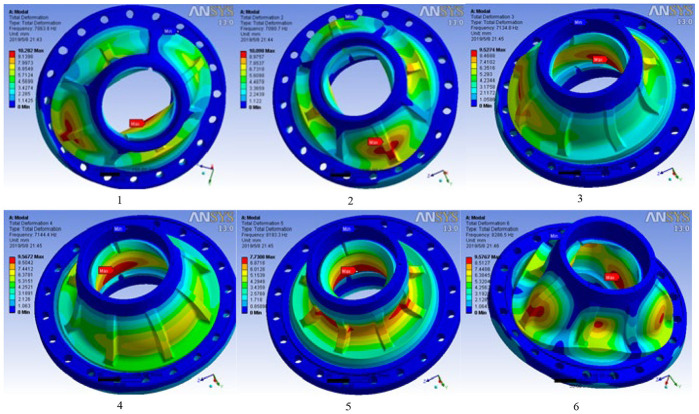
Six-order vibration frequencies.

From Figure 3, it can be seen that the maximum stress occurs at the flange surface and the rabbet of steel ring, which is 134.51 MPa (Figure 3(a)), less than the yield strength of material QT500-10, 360 MPa, and the maximum displacement value of hub is 0.01254 mm (Figure 3(b)). Furthermore, the safety factor of the hub is analyzed as shown in Figure 4. The minimum safety factor of the hub is 4.6467 (Figure 3(c)), which is larger than the minimum safety factor of 2.0–2.5 required by the hub. It occurs at the flange surface and the rabbet of steel ring. Therefore, the hub can be lightweight design, improve the structure size of the hub, and reduce the quality of the hub.

### Modal analysis

Modal analysis^18^ is a linear dynamic response process. The objective of modal analysis in structural mechanics is to determine the natural modal shape and frequency of an object or structure during free vibration, so as to enhance the natural frequency of structural characteristics and change the structure. After analysis and simulation by software, the corresponding six-order vibration frequencies can be obtained, and the cylindrical distribution of hub vibration frequencies is shown in Figure 4.

**Figure 5.**
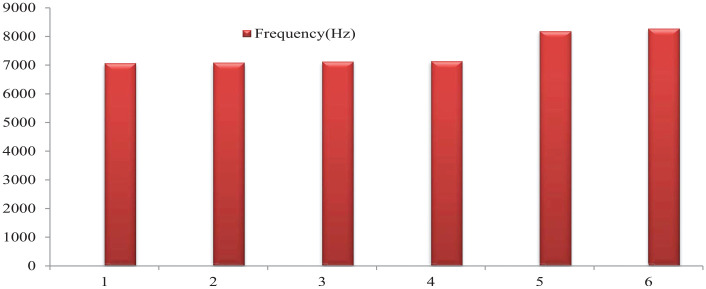
Cylindrical distribution of hub vibration frequency.

As can be seen from Figure 4, the frequencies of each order of the hub model are 7063.8–8286.5 Hz (Figure 5), which is much larger than the frequencies of 50 Hz when the hub is designed and operated. Therefore, there is no rigid motion of the hub, that is, resonance will not occur, which ensures that the hub will not be damaged by resonance.

### Fatigue life analysis

Hub fatigue belongs to high cycle fatigue,^19^ and its life cycle number *N* should be more than 10^6^: $N=10^{6}$ .

Stress value *σ* at cycle number $10^{6}$

$$\sigma =\sigma_{r}\sqrt[{m}]{\frac{N_{0}}{N}}=206\times \sqrt[{11.7662}]{\frac{1.78\times 10^{7}}{10^{6}}}\mathrm{MPa}=263.111\;\mathrm{MPa}$$

Because the hub is subjected to unidirectional unstable impact load, the fatigue factor *S* of the hub structure should be satisfied to ensure that the hub will not be damaged

$$S=\frac{\sigma }{\sigma_{\max}}>1$$

In the above-mentioned static analysis, the known maximum stress value $\sigma_{\max}$ of hub is 134.51 MPa

$$S=\frac{\sigma }{\sigma_{\max}}=\frac{263.111}{134.51}=1.956$$

In the actual service process, the stress value of the hub will change at any time during the movement, which cannot be applied to the *σ–N* curve conditions. Therefore, this article uses Miner’s rule^20^ to approximate the explanation. Miner’s rule is one of the most widely used cumulative damage models in fatigue analysis. If there are *k* different stress levels, under the *i*th stress $\sigma_{i}$ , the number of fatigue failure cycles on the *σ–N* curve of the material is $N_{i}$ , and the stress $\sigma_{i}$ is the corresponding number of cycles $n_{i}$ . The damage score *E* is

$$\sum_{i=1}^{k}\frac{n_{i}\times \sigma_{i}}{N_{i}\times \sigma_{i}}=E$$

where *E* is a fraction of the life expended by exposure to cycling at different stress levels. Usually, when the damage score is 1, the parts will fail. But in practice, *E* usually takes 0.7.

The fatigue coefficient used safely in actual structures is *S*’

S′=S×E=1.956×0.7=1.369>1

Through the above theoretical calculation, it is known that the fatigue life of the hub meets the safety use standard. The accuracy will be verified by software below.

### Finite element fatigue analysis

**Figure 6.**
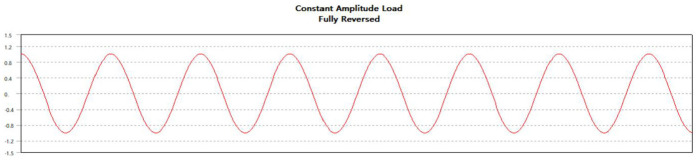
Random fatigue coefficient.

The mesh element partition and boundary constraint conditions of hub are the same as those of static analysis. The random fatigue coefficient generated by software simulation is shown in Figure 6.

In this article, the number of life cycles of hub is set as $10^{6}$ , the value of fatigue strength coefficient is 1, and the fatigue notch coefficient $K_{f}$

$$K_{f}=\frac{\sigma_{\max}}{\sigma_{r}\left(\mathrm{Fatigue}\;\mathrm{Limit}\;\mathrm{Value}\;\mathrm{under}\;\mathrm{Cyclic}\;\mathrm{Number}\;N_{0}\right)}=\frac{134.51}{206}=0.65$$

**Figure 7.**
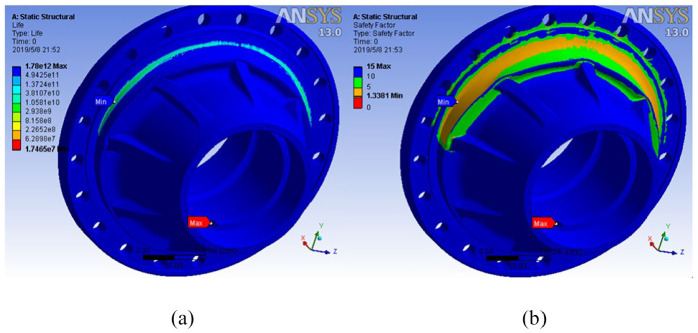
(a) Number of life cycles and (b) fatigue coefficient for safe use of structures.

Based on fatigue analysis, the number of life cycles and the fatigue coefficient of safe operation of the hub can be obtained (Figure 7). It can be seen that the hub is liable to be damaged and fractured when the hub is installed at the rabbet and flange surface after cycling for a certain number of cycles under load.

The minimum life cycle number $N_{\min}=1.7465\times 10^{7}$ is similar to the turning point cycle number $N_{0}=1.78\times 10^{7}$ and much larger than the hub life cycle number $N=10^{6}$ .

At the same time, the actual fatigue safety factor of the hub is 1.3381 greater than 1, which is close to the theoretical value *S*′ = 1.369, which verifies the reliability of fatigue simulation analysis.

## Results and discussion

### Lightweight design

**Figure 8.**
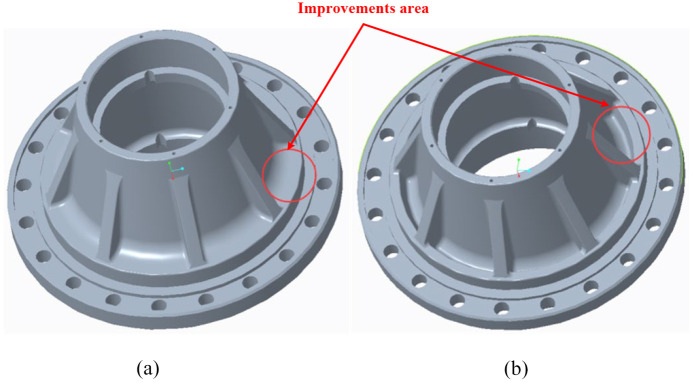
Comparison of three-dimensional models for lightweight design: (a) before lightweight design and (b) after lightweight design.

From the above static, modal, and fatigue life analysis, the necessary structure of the hub is obtained, which is the hub mounting rabbet, flange, and tire bolt hole. These structures bear large loads and torques, require high strength and stiffness, and have assembly constraints on tire bolt holes and tires, so it is not easy to modify their size and structure. Therefore, we should find out the non-important structure of the hub. Non-important structure refers to the structure that has little influence on the stress and performance of the hub. Based on the design and manufacturing experience of engineering personnel, there is no significant effect on the distribution of the whole hub structure after loading at the step between the hub mounting rabbet and the hub entity. In the lightweight design of hub, the non-important structure is removed from the original hub, so as to reduce the weight of hub and achieve the purpose of lightweight. The local parameters and three-dimensional model of lightweight hub are shown in Figure 8.

Of course, this may not be optimal, but it is better than the original sample. Lightweight design is a process of continuous improvement. We are constantly improving it based on the combination of theory and practical experience.

### Results

**Figure 9.**
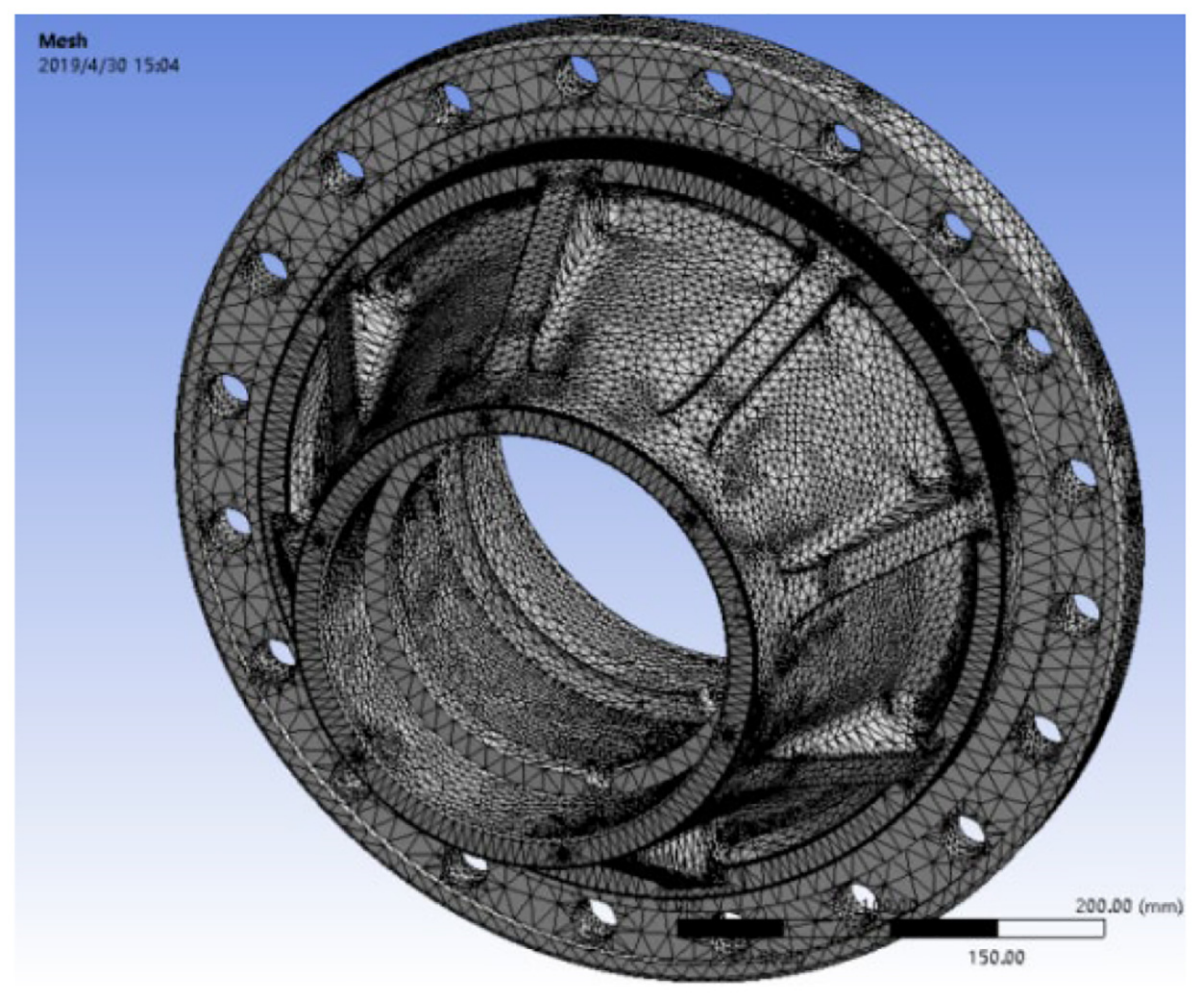
Improved hub mesh diagram.

After lightweight design, the hub is meshed, and the number of nodes is 532,837, with 31,762 entity units (Figure 9).

The relevant indicators of wheel hubs before and after lightweight design are shown in Table 1.

**Table 1.** Contrast before and after lightweight design.

Parameters	Before lightweight design	After lightweight design
Volume $\left(mm^{3}\right)$	$1.206\times 10^{6}$	$1.1392\times 10^{6}$
Mass (kg)	85.626	80.885
Maximum displacement (mm)	0.01254	0.025477
Maximum stress (MPa)	134.51	174.19
Safety factor	4.6467	3.588
Reduce mass (%)	–	5.537

By modifying the lightweight design of the hub structure, the mass of the hub is reduced by 4.741 kg and the weight is reduced by 5.537%. The minimum safety factor after improvement is 3.588, which is larger than the minimum safety factor of hub 2.0–2.5. The maximum stress value is 174.19 MPa, which is less than the yield strength of 360 MPa. Therefore, the lightweight hub meets the design requirements.

### Discussion

In order to further verify whether the lightweight hub performance conditions are satisfied, the modal and fatigue life are used to simulate.

Comparisons of the six-order natural frequencies of the front and rear wheels before and after lightweight are shown in Table 2.

**Table 2.** Comparisons of the six-order natural frequencies.

Order	1	2	3	4	5	6
Pre-optimization frequency (Hz)	7063.8	7080.7	7134.8	7144.4	8193.3	8286.5
Optimized frequency (Hz)	6897.0	6913.6	7051.2	7062.7	7921.2	7933.4
Change rate (%)	2.36	2.36	1.17	1.14	3.32	4.26

**Figure 10.**
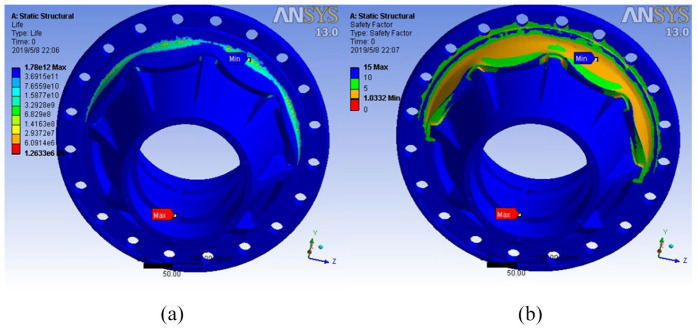
(a) Life cycle times and (b) safety factor of lightweight hub model.

From Table 2, the minimum natural frequency of lightweight hub is 6897.0 Hz, which is still larger than the designed operating frequency of the hub 50 Hz. The frequency changes little before and after the improvement, and the stability of the hub does not change greatly, and resonance phenomenon does not occur. The life cycle times and safety fatigue coefficient of lightweight hub model are shown in Figure 10.

Figure 9 shows that the minimum number $N_{\min}$ of life cycles of the lightweight rear hub model is $1.263\times 10^{6}$ , which is larger than the preset fatigue life cycle value $N=10^{6}$ . It should be noted that the maximum service life of the special axle of the hub is $0.143\times 10^{6}$ , which is far lower than $10^{6}$ . Therefore, in the design, we set the safety factor of 1, which is enough to meet the actual use of the axle. The minimum value of the safety fatigue factor *S*′ of the improved hub structure is 1.0322 larger than 1, which meets the requirements of the conditional design.

Lightweight design is of great significance for manufacturing enterprises facing comprehensive competition of resources,^21^ environment, and cost. Continuous improvement of modeling analysis and lightweight design for an axle hub considering stress and fatigue life is necessary for the sustainable development ability of the enterprises.

## Conclusion

Lightweight parts play an important role in improving automotive performance and reducing costs for sustainable development of manufacturing enterprises. In this article, the static, modal, and fatigue life of the hub are analyzed and simulated. Based on the analysis results, the local structure of the hub is improved with lightweight, and its rationality and correctness are verified. After lightening, the hub quality is 5.537% lower than before. The performance parameters meet the design scope and achieve the expected lightening results. The structure of wheel hub is improved to save materials, reduce production costs, and shorten R&D and design cycle.

Manufacturing enterprises are facing increasing pressure from environment, economy, and society. It is effective and significant to solve these problems from the technical level. This article has important reference and practical significance for lightweight design of automobile parts. It provides methods and technical support for the sustainable development of manufacturing enterprises.
